# Supplementary material for: Targeting PI3K-gamma in myeloid driven tumour immune suppression: a systematic review and meta-analysis of the preclinical literature
Source: Cancer Immunol Immunother. 2024 Aug 6;73(10):204. doi: 10.1007/s00262-024-03779-2 (PMC11303654; doi:10.1007/s00262-024-03779-2)
Supplement: Supplementary file 2 — Supplementary file2 (PPTX 51 kb) [file 262_2024_3779_MOESM2_ESM.pptx]

## Slide 1
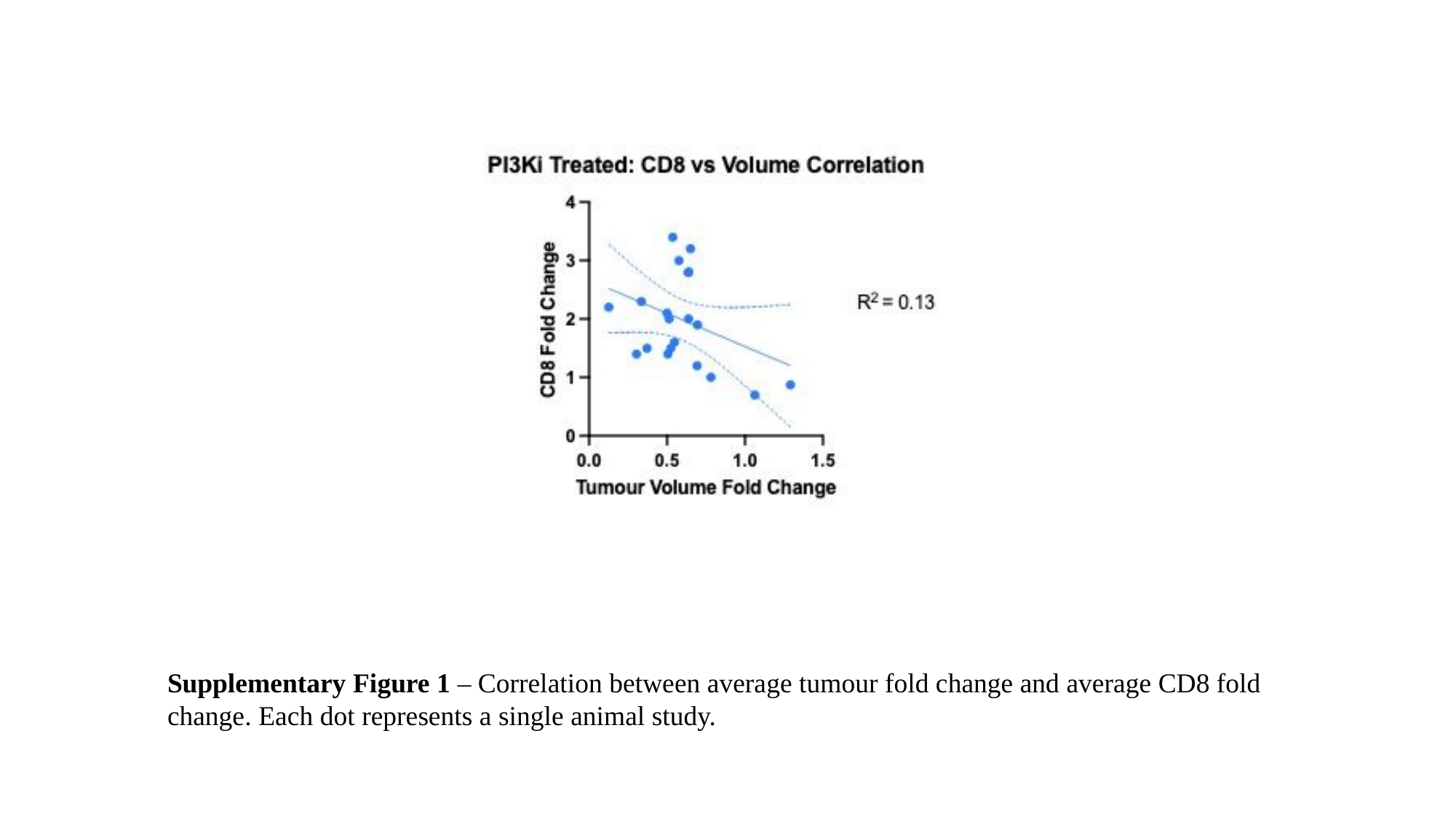

Supplementary Figure 1 – Correlation between average tumour fold change and average CD8 fold change. Each dot represents a single animal study.
