# Supplementary material for: Targeting PI3K-gamma in myeloid driven tumour immune suppression: a systematic review and meta-analysis of the preclinical literature
Source: Cancer Immunol Immunother. 2024 Aug 6;73(10):204. doi: 10.1007/s00262-024-03779-2 (PMC11303654; doi:10.1007/s00262-024-03779-2)
Supplement: Supplementary file 5 — Supplementary file5 (DOCX 408 kb) [file 262_2024_3779_MOESM5_ESM.docx]

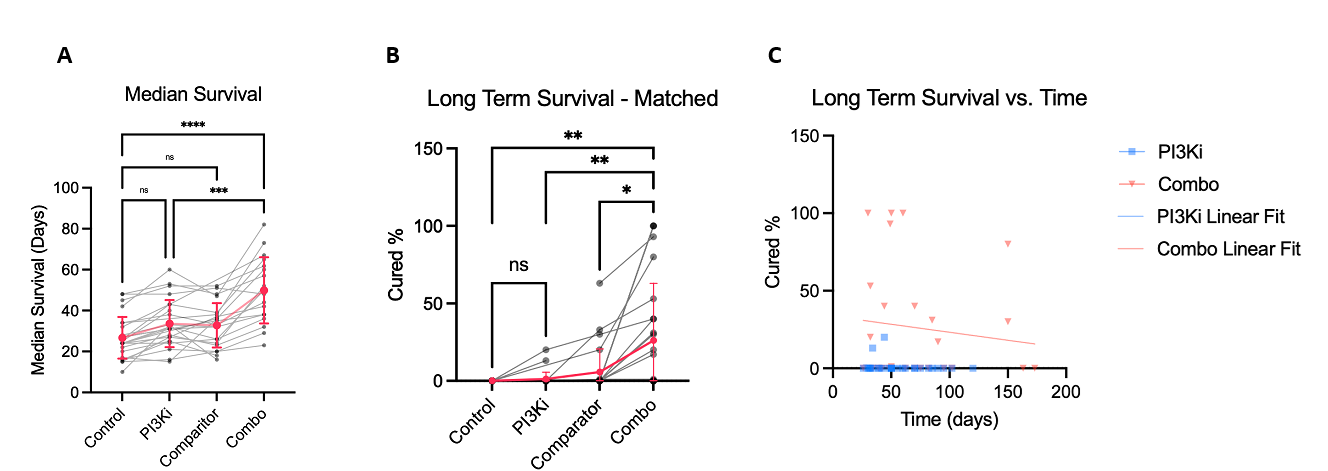


**Figure 3** – Summary of suvival data for groups receiving treatment as indicated. A, median oversall survival according to treatment group. B, rates of tumour regression (cure) according to treatment group. C, plot of cure rate over time with linear modelling according to PI3Kγ inhibitor monotherapy or combination treatment.

**Figure 4 –** Heat maps showing relative change of immune cell populations reported by study. A, Immune fraction changes extracted from studies reporting the effect of PI3Kγ inhibition alone. B, Immune fraction changes extracted form studies comparing combination therapy to control. Row label refer to the study number as seen in Table 1.

**Figure 5 –** Changes in CD8 T cells according to treatment group. A, CD8 T cell fold change in groups receiving PI3Kγ inhibitor monotherapy (blue) and combination therapy (red). B, CD8 T cell fold change according to type of combination therapy.
